# Comment on “Nationwide randomised trial evaluating elective neck dissection for early stage oral cancer (SEND study) with meta-analysis and concurrent real-world cohort”

**DOI:** 10.1038/s41416-020-0982-8

**Published:** 2020-07-16

**Authors:** Abhijeet Singh, Anand Subash, Piyush Sinha

**Affiliations:** 1 Department of Surgical Oncology, AIIMS, Hrishikesh, Uttarakhand India; 2 Department of Head and Neck Surgical Oncology, HCG Cancer Centre, Bangalore, Karnataka India; 3 Department of Head and Neck Surgical Oncology, Medanta Hospital, Lucknow, Uttar Pradesh India

**Keywords:** Surgical oncology, Oral cancer

We would like to congratulate the authors for a well-planned study (multicentric, randomised control trial with a real-world cohort arm+ meta-analysis).^1^ We believe that to truly evaluate the role of elective neck dissection (END) on survival outcomes, the role of other confounding factors like additional adjuvant treatment must be eliminated and this may not always be practical.

Positive cut margins and close margins are inherent risk factors for increased loco-regional recurrences. It is interesting to note that in the current study, less than half of the patients had clear margins in both the phases of the study. Yet, the overall recurrence rates were comparable to the Mumbai trial^2^ that had a margin positivity rate of about 3%. If the authors may elaborate on this difference, it could shed light on the debate that tumour biology related to smoked and smokeless tobacco across geographical areas is actually different. This could also underscore the need for a region-specific guideline than a global unified guideline.

Positive and often close margin with high-risk features are indications for adjuvant chemoradiotherapy, and this has been shown to have improved disease-free survival and overall survival.^3^ An increased rate of close margin in both the phases of this study mandated that an unexpectedly higher number of patients were subjected to multi-modality treatment. This in itself could be a bias in assessing survival and recurrence-free outcomes. Thus, the true survival benefit of elective neck dissection over observation may be obscured in the current study. Furthermore, a subset analysis excluding the pN+ cases might add more robustness to the study, which the Mumbai trial also failed to analyse.

An absorbing finding was made in the Mumbai trial, when the true negatives in the elective neck dissection arm were compared with the true negatives of the observation arm. The overall survival in the two groups was comparable, undermining the beneficial role of END in these cases. The authors may probably do a similar analysis to understand if there exists any variation across continents, considering the different risk factors for oral cavity malignancy.

Elective neck dissection probably eliminates the micrometastasis (MM) and isolated tumour cells (ITC).^4^ This has not been studied in any of the studies included in the meta-analysis, including the SEND trial. This may be the reason why END has shown to have reduced risk of regional recurrence and better survival outcomes. This is bespoken as both the Mumbai trial and SEND trial showed that END had no bearing on distant metastasis. To validate this point, all future trials should look at serial sectioning of pN0 lymph nodes with immunohistochemistry for MM and ITC.

The authors of SEND trial reported a weightage of ~21% in their final meta-analysis. We are in complete agreement with the conclusion that END offers a definitive survival benefit. When only the Mumbai trial and SEND trial were included in the analysis, this benefit was even more pronounced. The execution of the current trial could have been better with impactful conclusions, provided the need for multi-modality treatment was minimised. Nevertheless, SEND trial adds to level 1 evidence and merits the fame of a practice-changing trial.

## Data Availability

Not available.
